# Supplementary material for: Circuit-Based Design of Microfluidic Drop Networks
Source: Micromachines (Basel). 2022 Jul 16;13(7):1124. doi: 10.3390/mi13071124 (PMC9315978; doi:10.3390/mi13071124)
Supplement: Supplementary file 1 [file micromachines-13-01124-s001.zip › micromachines-1786054-supplementary.pdf]

micromachines

Supplementary Materials: Circuit-Based Design of Microfluidic Drop Networks

Nassim Rousset, Christian Lohasz, Julia Alicia Boos, Patrick M. Misun, Fernando Cardes and Andreas Hierlemann

Supplementary figures

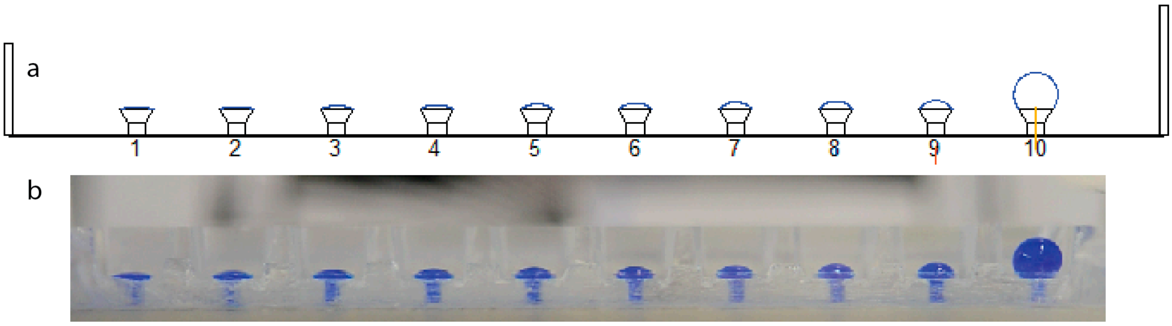

Figure S1. Modeling (a) and experimental (b) results of the operation of the scalable microfluidic chip elaborated further in the manuscript text. The figure is shown right before failure of the downstream standing drop #10 due to excessive pressure. The outlet, on the right, was blocked off, allowing liquid to accumulate in the last drop and causing a drop crash.

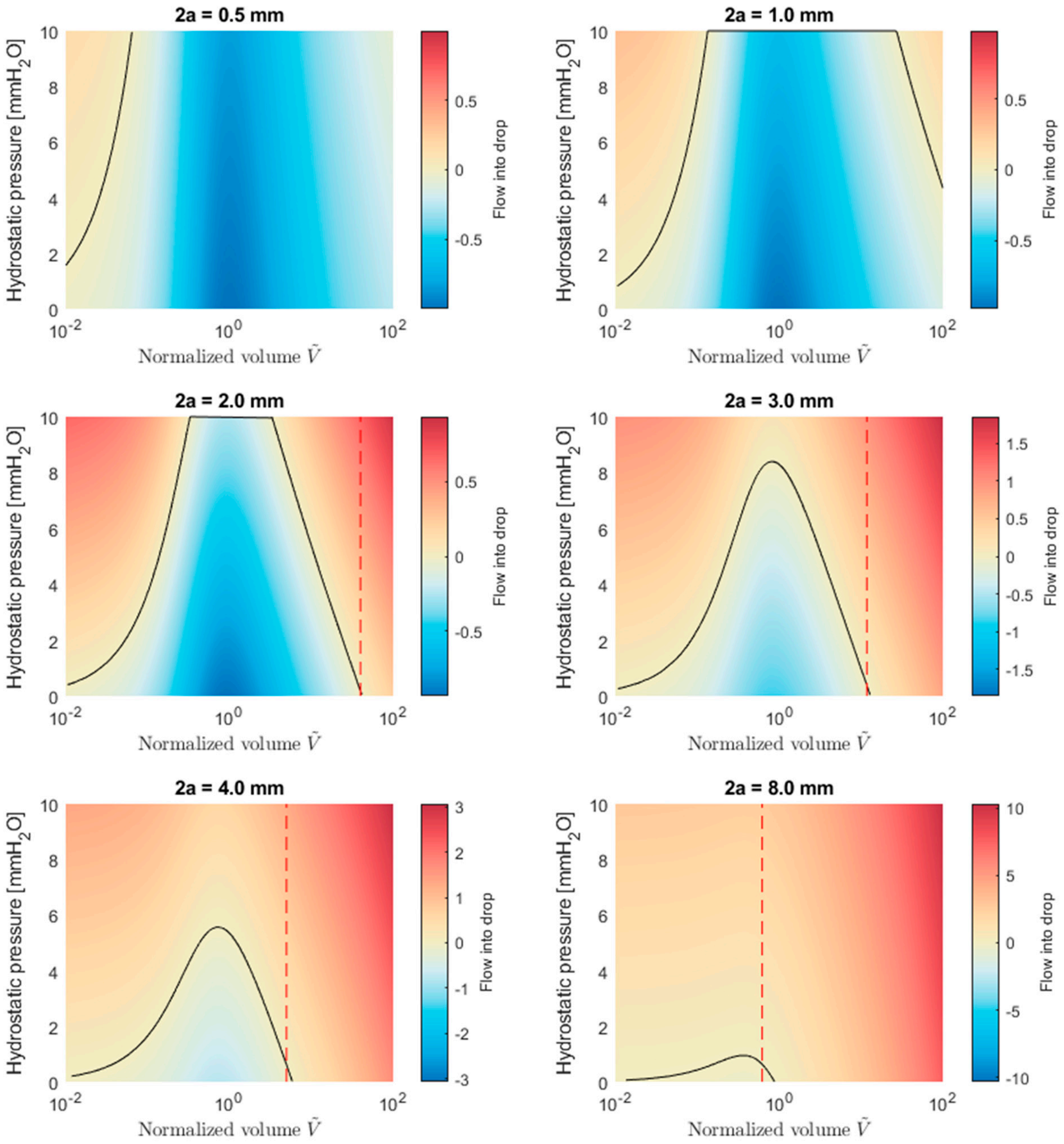

Figure S2. Phase diagram of hanging drops of various aperture diameters (2a). The critical normalized drop volume over which drop crash occurred is plotted as a dashed red line.

Circuit design strategy

In this section, we present details of the equations required to model circuits in a non-matrix form. We outline here a protocol that allows to draw the circuit without forgetting elements.

1. Define atmospheric pressure;
2. Start from a source:
    a. If there is a clear inlet and outlet, e.g., for flow-through devices, start with the inlet;
    b. If there is no clear inlet and outlet, e.g., for recirculating devices, choose an arbitrary source;

3. Following the intended direction of flow, work your way through the microfluidic design, node-by-node, replacing each element with its appropriate scheme. A node is defined as a point, where the flow can "choose" a path:
    a. Define an unknown pressure variable p_i at each node;
    b. Define a known capillary pressure p_{ci} and/or hydrostatic pressure p_{gi} at each drop or reservoir ALI nodes;
    c. Define a known resistance R_i between each node;
    d. Define an unknown flow rate Q_i and the intended direction of flow with an arrow through each element;
    e. If applicable, define a known inlet Q_{in} and/or outlet Q_{out} flow rate;
4. Terminate each fluid source, sink, or free surface by connecting it to atmospheric pressure. The importance of defining the atmospheric pressure is detailed in Section 2.3 "Atmospheric pressure".

As an example, we show the known and unknown variable definition, and set of equations written out for $N = 3$. First, we define the known variables:

$$p_{g-in}, p_{g-out}, p_{c1}, p_{g1}, p_{c2}, p_{g2}, p_{c3}, p_{g3}, R_{in}, , R_{c1}, R_{c2}, R_{c3}, R_1, R_2, R_3 = R_{out}$$

Then, we define the unknown variables:

$$p_1, p_2, p_3, Q_{c1}, Q_{c2}, Q_{c3}, Q_{in}, Q_1, Q_2, Q_3 = Q_{out}$$

With 10 unknown variables, we can write 10 orthogonal equations:

1. $p_{g-in} - Q_{in}R_{in} = p_1$
2. $p_1 - Q_1R_1 = p_2$
3. $p_2 - Q_2R_2 = p_3$
4. $p_3 - Q_3R_{out} = p_{g-out}$
5. $p_1 - Q_{c1}R_{c1} = p_{g-1} + p_{c-1}$
6. $p_2 - Q_{c2}R_{c2} = p_{g-2} + p_{c-2}$
7. $p_3 - Q_{c3}R_{c3} = p_{g-3} + p_{c-3}$
8. $Q_{in} = Q_1 + Q_{c1}$
9. $Q_1 = Q_2 + Q_{c2}$
10. $Q_2 = Q_{out} + Q_{c3}$

These equations were used to define the matrix in equation (7).

Contact-angle hysteresis

In this section, we elaborate on the complex dynamic of air-liquid-solid interfaces in movement. Wetting rough surfaces will lead to a difference in contact angles, when the air-liquid-solid tripoint of a drop moves into the air phase or into the liquid phase. This effect is called contact-angle hysteresis. The hysteresis can be quantified as an activation energy by the changes in interfacial area [20]. This contact angle hysteresis, plotted in Figure S3, results in a smoothing of the movement of drops, where the advancing or receding air-liquid tripoints will vary as a function of drop movement [19]. This hysteresis results in the oscillatory behavior of drop volume when actuated with a needle-type valve that is used to control the drop height (result in Figure 11).

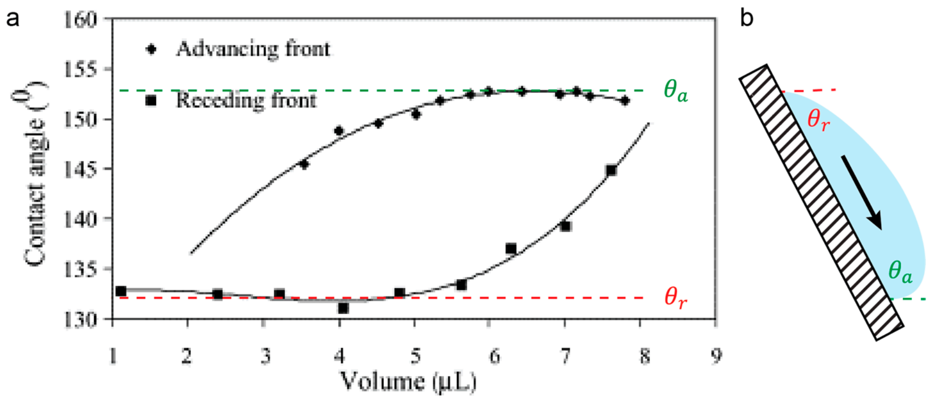

Figure S3. Advancing and receding contact angle measurements of a drop. The plot shows the hysteresis loop for the apparent contact angle and the drop volume. The advancing and receding contact angles were θ_a and θ_r. The difference between these two contact angles $\Delta\theta$ was used to determine the advancing and receding contact angles for other materials, where $\theta_a = \theta + \Delta\theta/2$ and $\theta_r = \theta - \Delta\theta/2$. The figure was reproduced and annotated with angles with permission from Elsevier [19]. (b) Schematic representation of an advancing (hashed green) and receding (hashed red) air-liquid-solid tripoint for a sliding drop.

Filling or emptying reservoirs

Modeling the volumes in our standing-drop compartments required modeling of the filling and emptying of the compartment dead volume (Figure 6). The simplified cross-sectional geometry of the standing-drop compartment is shown in Figure S4.

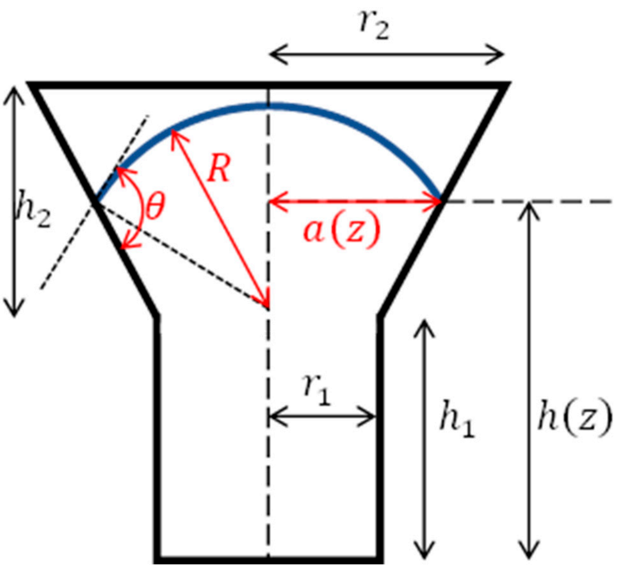

Figure S4. Geometric properties of the standing-drop compartments described by Figure 6. Each compartment volume consisted of a cylinder (radius r_1, and height h_1), capped with a truncated cone (small radius r_1, large radius r_2, and height h_2), capped with a spherical cap (aperture r_2, contact angle θ, and ALI curvature R). As the compartment filled or emptied, the air-liquid interface was described by its height $h(z)$ and its aperture $a(z)$.

An important geometric parameter was the relative angles of the cone and cylinder.

$$\theta_{\text{geom}} = \begin{cases} \text{atan}\left(\dfrac{h_2}{r_2 - r_1}\right) \to \text{Cone} \\ \dfrac{\pi}{2} \to \text{Cylinder} \end{cases}$$

This geometric parameter was used to find the contact angle of the advancing and receding fronts. The angle change from the cylinder to the cone caused a change in the contact angle of the air-liquid-solid tripoint, changing filling dynamics.

$$\theta_{a|r} = \theta_{\text{geom}} + \text{asin}\left(\frac{a(z)}{R_{a|r}}\right) \to R_{a|r} = \frac{a}{\sin\left(\theta_{a|r} - \theta_{\text{geom}}\right)} \to \begin{cases} R \geq a(z) \to \text{Convex spherical cap} \\ R \leq -a(z) \to \text{Concave spherical cap} \end{cases}$$

The liquid phase could be fully described with the following parameters:

- Advancing/Receding angle: $\theta_{a|r}$
- Advancing/Receding capillary curvature: $R_{a|r}(z) = \frac{a(z)}{\sin\left(\theta_{a|r} - \theta_{\text{geom}}(z)\right)}$
- Advancing/Receding spherical cap volume
    - $V_a = \frac{2\pi}{3} R_{a|r}(z)^3 - \frac{\pi}{3}\left(2R_{a|r}(z)^2 + a(z)^2\right)\sqrt{R_{a|r}(z)^2 - a(z)^2}$
    - $V_{a|r} = \frac{\pi}{3} a(z)^3 \left(2\csc^3\left(\theta_{a|r} - \theta_{\text{geom}}(z)\right) - \left(2\csc^2\left(\theta_{a|r} - \theta_{\text{geom}}(z)\right) + 1\right)\cot\left(\theta_{a|r} - \theta_{\text{geom}}(z)\right)\right)$
- Advancing/Receding total volume
- Volume in the cone:
    - $V_{co} = \frac{\pi}{3}\left(r_1{}^2 + r_1 a(z) + a(z)^2\right)\frac{a(z) - r_1}{r_2 - r_1} h_2 = \frac{\pi}{3}\frac{a(z)^3 - r_1{}^3}{r_2 - r_1} h_2 = \frac{\pi}{3}\left(a^3 C_{co} + D_{co}\right)$

The following Figure S5 describes the filling dynamics for hydrophobic (contact angle > $\pi/2$) and hydrophilic (contact angle < $\pi/2$)

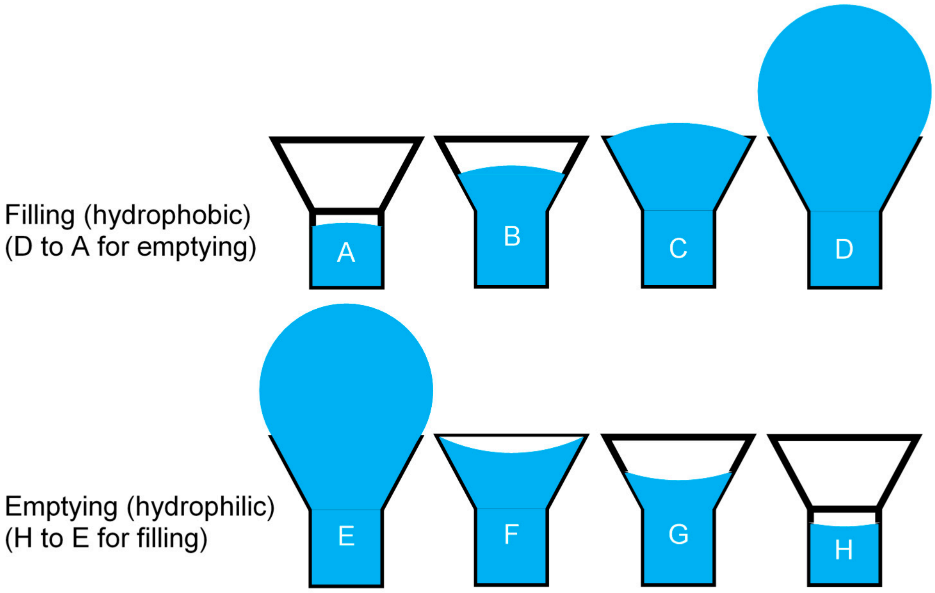

Figure S5. Filling and emptying dynamics of a standing-drop port for a hydrophobic advancing capillary front or for a hydrophilic receding capillary front. The filling and emptying steps are: cylinder (a, h), cone (b, g), spherical cap (c, f), and spherical cap crash limit (d, e) where an error is caused.

We summarize calculating steps for filling a standing drop in Table S1 and for emptying a standing drop in Table S2. The tables refer to Supplementary Figure 3 with the filling and emptying steps. The filling featured an unwanted "ERROR" step, whereby the flow filled the standing drop past the critical capillary length where the drop crashed. This is the condition shown in Figure S1. The emptying featured an unwanted "ERROR" step whereby the flow emptied the cylinder past its minimal volume, which meant that the device dried out.

Table S1: Calculating volumes at each filling step (advancing capillary front)

Copyright: © 2022 by the authors. Licensee MDPI, Basel, Switzerland. This article is an open access article distributed under the terms and conditions of the Creative Commons Attribution (CC BY) license (https://creativecommons.org/licenses/by/4.0/).

Filling	General	Cylinder	Cone	Spherical cap
(A) Cylinder	Flow fills cylinder until cone	Determined by flow (variable)	Null	Determined by advancing contact angle and cylinder radius (constant)
(B) Cone	Flow fills cone & adds cap radius until full	Full	Determined by flow (variable)	Determined by advancing contact angle and effective cone top radius (variable)
(C) Cap	Flow fills cap until Bond number (ERROR)	Full	Full	Determined by flow (variable) – changes capillary radius
*Switch from emptying	Flow fills cap only until advancing contact angle	Same as emptying	Same as emptying	Determined by flow (variable) – changes capillary radius

Table S2: Calculating volumes at each emptying step (receding capillary front)

Emptying	General	Cylinder	Cone	Spherical cap
(F) Cap	Flow empties cap until receding contact angle	Full	Full	Determined by flow (variable) – changes capillary radius
(G) Cone	Flow empties cone & reduces cap radius until cylinder	Full	Determined by flow (variable)	Determined by receding contact angle and effective cone top radius (variable)
(H) Cylinder	Flow empties cylinder until empty (ERROR)	Determined by flow (variable)	Null	Determined by receding contact angle and cylinder radius (constant)
*Switch from filling	Flow empties cap only until receding contact angle	Same as filling	Same as filling	Determined by flow (variable) – changes capillary radius

With the volume-calculating algorithms in Table S1 and Table S2, the equations describing each step can be written down. Table S3 shows these equations as a function of

the geometric parameters defined in Figure S4. The general algorithmic considerations for switching filling or emptying stages are annotated:

- "TUNE TIMESTEP": means that the time step allowing for a volume increase must be tuned to ensure that the volume increase does not overshoot the current stage change. For example, for an emptying cone, having calculated the emptying flow rate Q and with a time step Δt, if the volume increment $\Delta V = Q\Delta t$ empties the cone past its null volume, then the volume increment is limited ΔV_{max} and the time step $\Delta t_{max} = \Delta V/Q$.
- "ALL FLOW IS NULL!": means that the standing-drop compartment can no longer empty. This returns an error, meaning that the chip will dry out.
- "DROP BURST!": means that the standing drop exceeds the limit given by the critical capillary length and will tend to crash. This returns an error, meaning that the chip will leak.

Table S3: Volume-calculating equations for all filling/emptying steps

<table>
<tr><th></th><th>Stage</th><th>Cylinder</th><th>Cone</th><th>Spherical cap</th></tr>
<tr><td rowspan="4">Emptying</td><td>Cap</td><td>$\pi h_1 r_1^2$</td><td>$\frac{\pi}{3}({r_1}^2 + r_1 r_2 + {r_2}^2)h_2$</td><td>Find new R with inverse problem of:
$V = \frac{2\pi}{3}R^3 - \frac{\pi}{3}(2R^2 + {r_2}^2)\sqrt{R^2 - {r_2}^2}$
Until $R \geq R_r$ (TUNE TIMESTEP)</td></tr>
<tr><td>Cone</td><td>$\pi h_1 r_1^2$</td><td colspan="2">Find new $a(z)$ with inverse problem of:
$V = \frac{\pi}{3}(a^3(C_r + C_{co}) + D_{co})$
Until $a \leq r_1$ (TUNE TIMESTEP)</td></tr>
<tr><td>Cylinder</td><td>$\pi h(z) r_1^2$</td><td>0</td><td>$V_r = \frac{\pi}{3}{r_1}^3[-2\sec^3(\theta_r) + (2\sec^2(\theta_r) + 1)\tan(\theta_r)]$
Until $h(z) = 0$ (ALL FLOW IS NULL!)</td></tr>
<tr><td>*Switch from filling</td><td>Same as filling</td><td>Same as filling</td><td>Find new R with inverse problem of:
$V = \frac{2\pi}{3}R^3 - \frac{\pi}{3}(2R^2 + a(z)^2)\sqrt{R^2 - a(z)^2}$
Until $R = R_r$ (TUNE TIMESTEP)</td></tr>
<tr><td rowspan="4">Filling</td><td>Cylinder</td><td>$\pi h(z) r_1^2$</td><td>0</td><td>$V_a = \frac{\pi}{3}{r_1}^3[-2\sec^3(\theta_a) + (2\sec^2(\theta_a) + 1)\tan(\theta_a)]$
Until $h(z) = h_1$ (TUNE TIMESTEP)</td></tr>
<tr><td>Cone</td><td>$\pi h_1 r_1^2$</td><td colspan="2">Find new $a(z)$ with inverse problem of:
$V = \frac{\pi}{3}(a^3(C_a + C_{co}) + D_{co})$
Until $a = r_2$ (TUNE TIMESTEP)</td></tr>
<tr><td>Cap</td><td>$\pi h_1 r_1^2$</td><td>$\frac{\pi}{3}({r_1}^2 + r_1 r_2 + {r_2}^2)h_2$</td><td>Find new R with inverse problem of:
$V = \frac{2\pi}{3}R^3 - \frac{\pi}{3}(2R^2 + {r_2}^2)\sqrt{R^2 - {r_2}^2}$
Until $R > 4mm$ (DROP BURST!)</td></tr>
<tr><td>*Switch from emptying</td><td>Same as emptying</td><td>Same as emptying</td><td>Find new R with inverse problem of:
$V = \frac{2\pi}{3}R^3 - \frac{\pi}{3}(2R^2 + a(z)^2)\sqrt{R^2 - a(z)^2}$
Until $R = R_a$ (TUNE TIMESTEP)</td></tr>
</table>

Hanging-drop crash

A drop crash happens when the dimensions of a drop lead to an irrecuperable drop state. A dimensionless analysis of the relationship between the curvature of a spherical cap r of radius a and its volume V (per Table 2) highlights the behavior of crashing drops. The normalization is as follows:

- Normalized spherical cap volume $\tilde{V} = V/(2\pi a^3/3)$:
  - $\tilde{V} < 1$ is a stable drop smaller than a hemisphere;
  - $\tilde{V} = 1$ is a pseudo-stable drop equal to a hemisphere;

    - $\tilde{V} > 1$ is an unstable drop larger than a hemisphere;
- Normalized spherical cap curvature $\tilde{r} = r/a$:
    - $\tilde{r} < 1$ is impossible;
    - $\tilde{r} = 1$ is the minimum radius, giving a hemispherical drop;
    - $\tilde{r} > 1$ can either be larger or smaller than a hemisphere depending on the volume;
- Normalized capillary pressure $\tilde{p_c} = p_c/(2\gamma/a)$:
    - $\tilde{p_c} < 1$ can either be larger or smaller than a hemisphere depending on the volume;
    - $\tilde{p_c} = 1$ is the maximum pressure, when $r = a$;
    - $\tilde{p_c} > 1$ is impossible.

The chosen normalization allowed us to rewrite $r(V, a)$ from Table 2 as $\tilde{r}(\tilde{V})$ and $p_c(r)$ from equation (5) as $\tilde{p_c}(\tilde{r})$ in equation (S1):

$$\tilde{r}(\tilde{V}) = \frac{1}{8\tilde{V}}\left[1 + \left(2\tilde{V} + \sqrt{1 + (2\tilde{V})^2}\right)^{\frac{4}{3}} + \left(2\tilde{V} + \sqrt{1 + (2\tilde{V})^2}\right)^{-\frac{4}{3}}\right]$$
$$\tilde{p_c}(\tilde{r}) = \frac{1}{\tilde{r}} \tag{S1}$$

Combining these results gives us an expression for $\tilde{p_c}(\tilde{V})$ which allows us to establish a mathematical relation between capillary pressure and drop volume. The resulting equation (S2) is the black line "ALI pressure" plotted in Figure 4.

$$\tilde{p_c}(\tilde{V}) = 8\tilde{V}\left[1 + \left(2\tilde{V} + \sqrt{1 + (2\tilde{V})^2}\right)^{\frac{4}{3}} + \left(2\tilde{V} + \sqrt{1 + (2\tilde{V})^2}\right)^{-\frac{4}{3}}\right]^{-1} \tag{S2}$$

A hanging drop of a given volume and capillary pressure exists only on the curve given by $\tilde{p_c}(\tilde{V})$. In other words, as the volume of a hanging drop changes, it follows the black curve given in Figure 4.

Volume change is driven by the internal hydraulic pressure $p_n + p_g$. We looked at volume change strictly driven by hydrostatic pressure p_g, i.e., $p_n = 0$. As the drop volume changes, following equation (S2), its height, and, therefore, $p_g = \rho g h$, also changes. If internal pressure is less than capillary pressure, then the drop volume will decrease. If internal pressure exceeds capillary pressure, then the drop volume will increase. A drop will always settle to its stable point $p_c = p_n + p_g$.

We first derive the self-driven drop hydrostatic pressure by finding the drop height, where normalizing $h(a, V)$ from Table 2 with $\tilde{h} = h/a$, yields $\tilde{h}(\tilde{V})$:

$$\tilde{h}(\tilde{V}) = \left(2\tilde{V} + \sqrt{1 + (2\tilde{V})^2}\right)^{\frac{1}{3}} - \left(2\tilde{V} + \sqrt{1 + (2\tilde{V})^2}\right)^{-\frac{1}{3}} \tag{S3}$$

Thereafter, an added hydrostatic pressure was added by the height difference between the drop aperture and the highest liquid level exposed to atmospheric pressure Δh. This height difference can be due to the chip being out of level, or due to an open inlet on the upper side of the device. The normalized value of internal pressure under strictly hydrostatic pressure is plotted in Figure 4 (dashed black line). Equation (S4) is derived from the sum of the self-driven drop hydrostatic pressure of equation (S3) and the chip-driven hydrostatic pressure Δh.

$$\frac{p_n + p_g}{(2\gamma/a)} = \frac{\rho g a}{2\gamma}\left[a\left(2\tilde{V} + \sqrt{1 + (2\tilde{V})^2}\right)^{\frac{1}{3}} - a\left(2\tilde{V} + \sqrt{1 + (2\tilde{V})^2}\right)^{-\frac{1}{3}} + \Delta h\right] \tag{S4}$$

This equation varied as a function of the operational condition of the microfluidic chip, meaning the internal pressure $p_n + p_g$.
